# Effects of Long-Term Cold Stress on Growth Performance, Behavior, Physiological Parameters, and Energy Metabolism in Growing Beef Cattle

**DOI:** 10.3390/ani13101619

**Published:** 2023-05-12

**Authors:** Siyuan Wang, Qi Li, Jianhao Peng, Huaxin Niu

**Affiliations:** College of Animal Science and Technology, Inner Mongolia Minzu University, Tongliao 028000, China; wangsiyuanme@126.com (S.W.); liqi08061999@163.com (Q.L.); 18844182642@163.com (J.P.)

**Keywords:** beef cattle, long-term cold stress, growth performance, physiological parameters, blood indicators

## Abstract

**Simple Summary:**

Winter cold stress is a major environmental factor affecting cattle industry production in northern China. Determining the effects of prolonged cold stress on the relevant biological mechanisms in cattle can help develop effective mitigation measures. Therefore, this study evaluated the effects of long-term cold stress on growth performance, physiological mechanisms, blood biochemical, and stress hormones of Simmental crossbred bulls. Results indicated that the physiological behavior, digestive function, and contents of enzymes and hormones in the body changed in Simmental cattle during winter, which enhanced the body’s heat production to maintain the body temperature constant and ultimately led to impaired growth and development.

**Abstract:**

This study aimed to evaluate the effects of a long-term cold environment on growth performance, physiological behavior, biochemical blood indexes, and hormone levels in Simmental cattle. Thirty Simmental crossbred bulls (weight = 350 ± 17 kg, 13–14 months old) were selected for two trials at autumn suitable temperatures (A-ST) and winter cold temperatures (W-CT) (15 cattle per season). The results showed that compared with the A-ST group, dry matter intake (*p* < 0.05) and feed:gain (*p* < 0.01) of the W-CT group increased, while body weight (*p* < 0.01) and average daily gain (*p* < 0.01) significantly decreased. Long-term cold stress also increased lying time (*p* < 0.01), feeding time (*p* < 0.05), and pulse rate (*p* < 0.01) in the W-CT group, while the rumen volatile fatty acids content (*p* < 0.01) and apparent digestibility of nutrients (*p* < 0.05) were significantly decreased. In terms of blood indicators, long-term cold stress increased the concentrations of glucose, glucose metabolic enzymes, glucocorticoids, triiodothyronine, and tetraiodothyronine in the plasma of the W-CT group (*p* < 0.05), but the levels of triglycerides, β-hydroxybutyrate, propionate, insulin, and growth hormone were decreased (*p* < 0.01). In summary, long-term cold stress may inhibit the digestive function of Simmental cattle and enhance the body’s energy metabolism and stress hormone imbalance, ultimately damaging the normal growth and development of the body.

## 1. Introduction

Ambient temperature is an important external factor affecting the production performance of animals. The thermoneutral zone (TNZ) is defined as the range of ambient temperatures without regulatory changes in metabolic heat production or evaporative heat loss [1]. When constant temperature animals are in the TNZ, heat production is in relative equilibrium with heat dissipation, and growth and development performance reach optimal conditions [2]. When the external environment is too cold, heat dissipation is greater than heat production, at which point this thermal balance is disrupted, causing cold stress to the animal [3]. Exposure to cold temperatures activates peripheral receptors responsible for cold and hot sensation, and when signals are transmitted to the spinal cord, two types of reactions are produced through the sympathetic nervous system. Firstly, the innervation of blood vessels generates vasoconstriction and heat retention. An additional response is shivering, which increases heat production through the repetitive and rapid contraction of skeletal muscles. Furthermore, cold stress can also lead to changes in the levels of related hormones (such as thyroid hormone, insulin, adrenal hormone, etc.) in the body [4,5]. These mechanisms promote the production or retention of heat to avoid hypothermia.

Cold stress is a significant factor in the slow growth and increased mortality of animals, resulting in substantial economic losses to livestock worldwide, particularly in the long and cold northern hemisphere of winter [6]. At the same time, cold stress can be divided into acute cold stress and chronic (long-term) cold stress according to intensity and duration [7]. It has been reported that even long-term exposure to mild cold conditions in animals can produce physiological adaptations, including increased calorie production, appetite, and basal metabolic intensity, as well as changes in digestive function [8]. At present, as the production level increases, the breeding industry gradually changes to intensive production; however, due to the physiological habits of cattle, which still need to keep a certain place of activity, it is difficult to realize completely enclosed feeding [9]. Therefore, the lower ambient temperature in winter is still a concern for the cattle-raising industry. Furthermore, strong winter wind and snow induce more heat loss, which can exacerbate the cold stress of cattle. Studies have shown that dairy cows in northern China can reduce their milk production by up to 40% in cold winter environments, and the odds of mortality among calves aged 0–28 days were 2.09 times higher during winter compared to summer [10,11]. Kang et al. [12] reported that under long-term cold ambient conditions (long-term cold stress), the average daily gain (ADG), feed conversion rate, and immune function of Korean cattle significantly decreased. Nakajima et al. [13] also found similar results in Japanese black cattle, while Sasaki et al. [14] also found that the fertility rate of Japanese black cattle in winter is about 5% lower than that in autumn, and the calving interval in winter is the longest.

Inner Mongolia is located in northern China, with a temperate continental monsoon climate. The winter is cold and long; the annual average temperature is about 5.8 °C, the extreme minimum temperature is −34.6 °C, and the coldest month average temperature is −12.7–−16.1 °C. Simmental cattle is a major breeding breed in Inner Mongolia; its growth speed, production performance, reproductive performance, and immune function in winter directly affect the economic benefits of producers. However, there are currently few relevant studies on the effects of long-term cold stress on Simmental cattle. Therefore, this study aimed to understand the effects of cold ambient on Simmental cattle by examining their growth indexes, feed utilization, biochemical blood indexes, and glycolipid metabolism under long-term cold stress conditions, which could help improve animal welfare and reduce economic losses caused by cold stress.

## 2. Materials and Methods

### 2.1. Animals, Breeding Environment, and Facilities

The animal study protocol was approved by the Experimental Animal Welfare and Ethics Committee of the College of Animal Science and Technology of Inner Mongolia Minzu University (protocol code: No. 2020069). All animal experiments were performed in accordance with the national standard Guideline for Ethical Review of Animal Welfare (GB/T 35892-2018).

A total of 30 healthy Simmental hybrid bulls (weight = 350 ± 17 kg, 13–14 months old) raised in different seasons were used in the study. They were allocated into two groups: autumn suitable temperatures (A-ST) and winter cold temperatures (W-CT), with 15 cattle assigned to each season. The experiment was conducted at the Mengzhiyuan Cattle Farm in Tongliao, Inner Mongolia. During the autumn and winter trials, all animals were semi-housed in the same free space, provided the same total mixed ration (TMR) (Table 1), and drank freely. Ambient temperature and relative humidity were recorded every hour daily in the barn area using an electronic thermometer and hygrometer (GSP-8A, Jiangsu Jingchuang Electric Co., Ltd., Xuzhou, China). During the experiment, the daily maximum temperature, minimum temperature, average temperature, and relative humidity in autumn and winter were recorded, and the temperature and humidity index (THI) was calculated according to the following formula.

$$\mathrm{THI}=0.8\;\times \;\mathrm{ambient}\;\mathrm{temperature}+\left[\left(\%\;\mathrm{relative}\;\mathrm{humidity}\;÷\;\left.100\right)\;\times \;\left(\mathrm{ambient}\;\mathrm{temperature}\;-\;\left.14.4\right)\right.\right.\right]+46.4$$

### 2.2. Experimental Design

This experiment was conducted at Tongliao Mengzhiyuan Farming Professional Cooperative (43.4528° N, 121.16° E). Tongliao area has a temperate continental monsoon climate with an annual average temperature of 7.1 °C, annual precipitation of 350–450 mm, annual average sunshine hours of 2900 h, and an average frost-free period of about 140 d. Two experiments were respectively carried out in the autumn (10 September 2020 to 21 October 2020) and winter (22 December 2020 to 31 January 2021) seasons. Each trial lasted 42 days, with 15 cattle divided into 3 replicates, each with 5 heads, each replicated a fence (10 m wide × 12 m long).

### 2.3. Feed Intake and Growth Performance

During the autumn and winter seasons experiment, all bulls were weighed prior to morning feeding on two consecutive days at the beginning (initial body weight, IBW) and at the end (final body weight, FBW) of the experiment. Provided and remaining TMR weights were weighed and recorded daily to determine dry matter intake (DMI). Average daily gain (ADG = (FBW − IBW)/42 d) and feed efficiency (FE = DMI/ADG) were calculated.

### 2.4. Behavior and Physiological Responses

The behavior of the bulls was observed on the 40th day after the experiment began. Each fence had a network real-time e-monitor (DPH-OP-100, Huawei Technologies Co., Ltd., Shenzhen, China) to view the activity of all bulls. Observations were recorded every 5 min with respect to whether the cattle in each fence were lying, standing, feeding, ruminating, walking, or drinking. Behavior was recorded continuously during a 24-h period. Hence, all behavior results were expressed as a percentage of the 24-h period [15].

On the 41st day of the experiment, 4 h after feeding, the rectal temperature was recorded using a digital thermometer (SureTemp^®^Plus 690, Welch Allyn, Skaneateles Falls, NY, USA) with an accuracy of 0.01 °C, placed at a distance of 9 cm inside the rectum for approximately 30 s. The respiratory rate per minute was recorded using a stopwatch, and then the pulse rate per minute was recorded at the bull’s caudal artery.

### 2.5. Ruminal Fluid Collection and Volatile Fatty Acids Analysis

On the 41st day of the experiment, after taking the physiological parameters, 200 mL of rumen fluid was taken with a stomach tube and vacuum pump. The first 100 mL of the ruminal samples were discarded to avoid contamination by saliva. Immediately, the ruminal pH was measured with a portable pH meter (PHB-4, Lei-ci Co., Ltd., Shanghai, China). Ruminal fluid samples were centrifuged at 16,000× *g* for 15 min at 4 °C, and the supernatant was kept at −20 °C. The ruminal samples were thawed and utilized to analyze VFA (GC 8890; Agilent Technologies Ltd., Palo Alto, CA, USA).

### 2.6. Apparent Digestibility of Nutrients

The apparent total digestive tract digestibility of dry matter (DM), crude protein (CP), neutral detergent fiber (NDF), and acid detergent fiber (ADF) was measured using acid-insoluble ash (AIA) as an internal standard. Feed and fecal samples were collected twice a day on the 39th, 40th, and 41st days, respectively. Following collection, samples were stored at −20 °C until further processing and analysis. Samples of feeds and feces were dried in a forced air oven (DGX-9053B, Fuma Experimental Equipment Co., Ltd., Shanghai, China) at 55 °C for 48 h and ground in a mill to pass a 1-mm screen for the determination of DM in the diet and feces. Dietary and fecal CP content was determined by a semi-automatic Kjeldahl system (KDN-BI, Shanghai Xinjia Electronics Co., Ltd., Shanghai, China). Determinations of NDF and ADF were carried out according to Van Soest et al. (1991) [16]. Then, the apparent digestibility of each nutrient was calculated using the following formula: a certain nutrient digestibility (%) = 100 − [100 × (B × C)/(A × D)], where A represents a kind of nutrient content in the feed, B represents the nutrient content in feces, C represents AIA content in the feed, and D represents AIA content in feces.

### 2.7. Blood Collection and Analysis

On the 42nd day of each trial period, approximately 10 mL of blood was collected by jugular puncture before morning feeding. Blood samples were immediately placed on ice after collection and then centrifuged at 3000× *g* for 15 min at 4 °C. Subsequently, the obtained plasma was immediately transferred to a polypropylene bottle and stored at −20 °C for further analysis.

Biochemical blood indicators included glucose (GLU), triglyceride (TG), non-esterified fatty acids (NEFA), and β-hydroxybutyrate (BHB), which were determined by an automatic biochemistry analyzer (Cobas c311, Roche, Basel, Switzerland) according to the instrument’s instructions. Propionate contents of blood were analyzed (GC 8890; Agilent Technologies Ltd., Palo Alto, CA, USA). Furthermore, the levels of acetyl-CoA (A-CoA, H331), citrate synthase (CS, A108), glucose-6-phosphatase (G-6-Pase, H580), pyruvate kinase (PK, A076), insulin (INS, H203), growth hormone (GH, H091), glucocorticoid (GC, H094), triiodothyronine (T3, H222), and tetraiodothyronine (T4, H223) in the plasma were assessed using commercial ELISA kits (Nanjing Jiancheng Bioengineering Institute, Nanjing, China) according to the manufacturer’s instructions, respectively. The ELISA results were obtained using a microplate reader (Varioskan LUX, ThermoFisher Scientific, Waltham, MA, USA).

### 2.8. Statistical Analysis

Statistical analysis was performed by using SPSS 27.0 software (International Business Machines Corporation, Armonk, NY, USA). Data were tested for normal distribution using the Shapiro–Wilk test. Student’s *t*-test was used to evaluate the difference in environmental parameters, growth performance, behavior, apparent digestibility of nutrients, and blood indexes between the A-ST group and the W-CT group. Experimental data are presented by means ± SEM. Differences were considered statistically significant at *p* < 0.05.

## 3. Results

### 3.1. Environmental Climate

**Figure 1:**
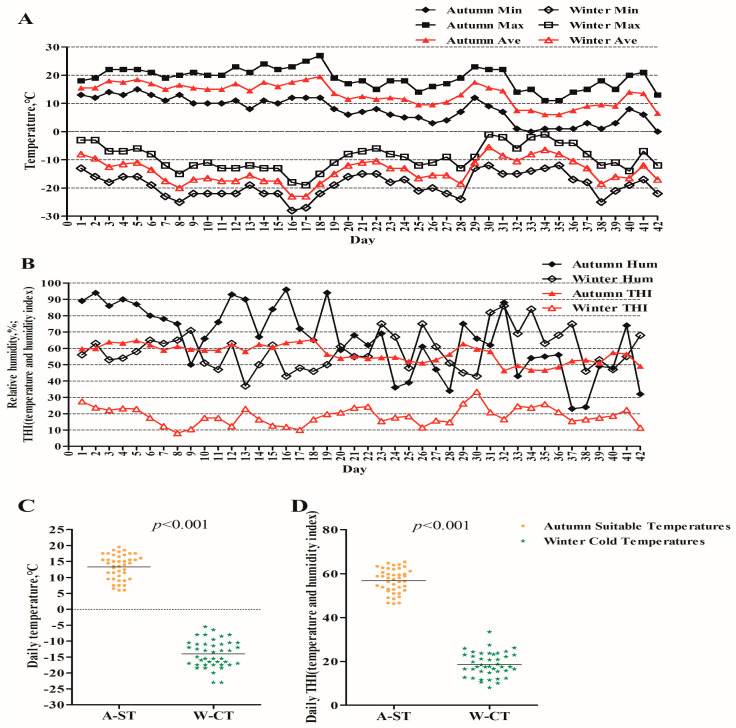
Experimental period ambient climatic conditions. (**A**) Daily temperature changes during the trial periods in autumn and winter; (**B**) Daily humidity and THI changes during the trial periods in autumn and winter; (**C**) Difference in mean temperature during the autumn and winter trial periods; (**D**) Difference in mean THI during the autumn and winter trial periods.

The temperature and humidity results of the autumn and winter test periods are shown in Figure 1. The maximum and minimum temperatures refer to the extreme temperatures reached during the test. The daily average temperature is used to evaluate the overall ambient temperature status during the test. Humidity and THI are the same as (Figure 1A,B). The mean temperature and mean THI in autumn were significantly higher than those in the winter (*p* < 0.01). The average temperature in the autumn was 13.29 °C, and the average THI was 56.77. During the winter experiment, the average temperature was −14.02 °C, lower than the critical temperature of TNZ (5 °C), and with an average THI of 18.60 (Figure 1C,D). Moreover, the lowest temperature was below −20 °C for 17 days during the winter experiment (Figure 1A).

### 3.2. Growth Performance

As shown in Table 2, at the beginning of the trial, there was no significant difference in IBW of Simmental cattle between the W-CT and A-ST groups (*p* > 0.05). After the experiment, the results showed that the FBW and ADG of Simmental cattle in the W-CT group were significantly lower than those in the A-ST group (*p* < 0.01), while the DMI (*p* < 0.05) and FE (*p* < 0.01) in the W-CT group were significantly higher than A-ST group.

### 3.3. Physiological Reactions and Behaviors

The behavior of Simmental cattle was monitored on the 40th day of the trial period. The results showed that, compared with the A-ST group, the lying time (*p* < 0.01) and feeding time (*p* < 0.05) of Simmental cattle in the W-CT group were increased, while standing time, walking time, and drinking time were significantly decreased (*p* < 0.01). The differences in standing rumination time and lying down rumination time between the two groups were not significant (*p* > 0.05) (Table 3).

**Figure 2:**
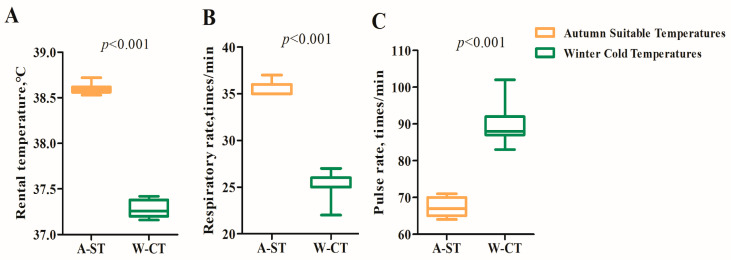
Effects of long-term cold stress on rectal temperature (**A**), respiratory rate (**B**), and pulse frequency (**C**) in Simmental bulls.

On the 41st day of the trial period, we measured the rectal temperature, respiratory rate, and pulse rate behavior of Simmental cattle. The results showed that the rectal temperature (Figure 2A) and respiratory rate (Figure 2B) of Simmental cattle in the W-CT group were significantly lower than those in the A-ST group (*p* < 0.01), while the pulse frequency was significantly higher than that in the A-ST group (*p* < 0.01) (Figure 2C).

### 3.4. Rumen Volatile Fatty Acids and Nutrients Apparent Digestibility

The results of rumen VFA measurements are shown in Table 4. The results showed that the contents of acetate, propionate, and total VFA in the rumen of Simmental cattle in the W-CT group were significantly lower than those in the A-ST group (*p* < 0.01), while the ratio of acetate to propionate was significantly higher than that in the A-ST group (*p* < 0.01). In addition, the difference in rumen pH between the two groups was not significant (*p* > 0.05).

Subsequently, we tested and analyzed the apparent digestibility of nutrients, and the results showed that the apparent digestibility of DM (*p* < 0.05), CP (*p* < 0.05), NDF (*p* < 0.01), ADF (*p* < 0.01), and energy (*p* < 0.01) were lower in the W-CT group than in the A-ST group (Table 5). This indicates that long-term cold stress in winter can significantly reduce the body’s nutrient digestion ability.

### 3.5. Blood Indexes

As known from Table 6, compared with the A-ST group, the levels of GLU in the plasma of the W-CT group were significantly increased (*p* < 0.01), while the levels of TG, BHB, and propionate in the plasma were significantly decreased (*p* < 0.01). The NEFA content was not significantly different between the two groups (*p* > 0.05).

**Figure 3:**
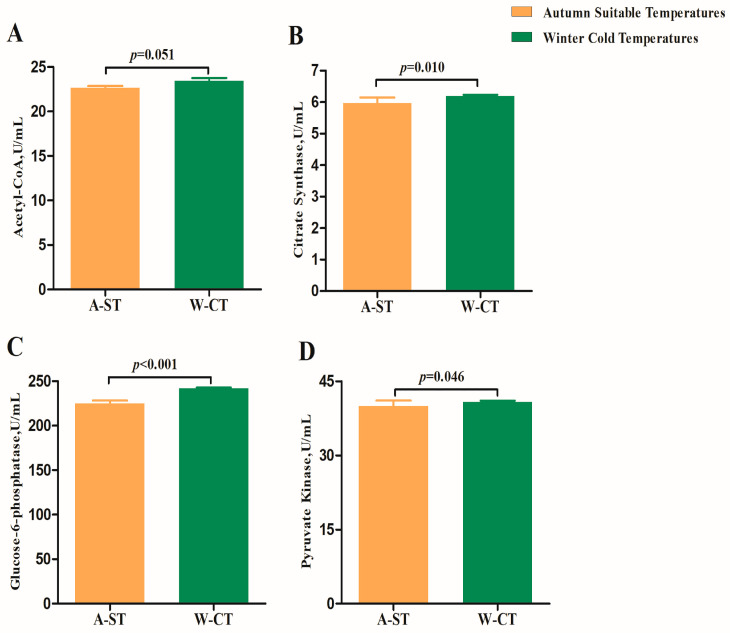
(**A**–**D**) Effects of long-term cold stress on plasma glucose metabolic enzymes in Simmental bulls.

Since the content of GLU in the plasma of the W-CT group was significantly increased, so we measured the concentration of glucose-metabolizing enzymes in the plasma. The results showed that the plasma concentrations of CS, G-6-Pase, and PK in the W-CT group were significantly higher than those in the A-ST group (*p* < 0.05), and the concentration of A-CoA did not change significantly (*p* > 0.05) (Figure 3).

The measurement results of stress hormones in plasma are shown in Table 7. The results showed that the plasma levels of INS and GH in the W-CT group were significantly lower than those in the A-ST group (*p* < 0.01), but the contents of GC (*p* < 0.01), T3 (*p* < 0.05), and T4 (*p* < 0.01) increased.

## 4. Discussion

Cold stress is an important factor restricting livestock production. In this study, we found differences in growth performance and physiological behavior of Simmental cattle between warm and cold seasons and analyzed changes in their rumen VFA, physiological blood indicators, and stress hormone levels. Revealed some physiological mechanisms of Simmental cattle’s resistance to cold stress and provided valuable information for improving the production efficiency of winter livestock and poultry raising.

### 4.1. Changes in Growth Performance and Physiological Behavior

Alterations in animal behavior occur in response to external environmental stress and can be divided into (i) behaviors to prevent hypothermia (including: increasing energy intake, adopting a ball-like position, basking in the sun and nest-building, etc.) and (ii) behaviors to prevent hyperthermia (including: reducing energy intake, increasing water consumption, shade-seeking and increasing respiratory rate, etc.) [17]. When cattle are under long-term cold stress, they also adopt corresponding behaviors to maintain a constant body temperature [4]. Generally, it is suggested that feeding behavior is the primary behavioral response of cattle under cold stress [18]. Theoretically, for every 1 °C decrease in ambient temperature, the maintenance energy required for beef cattle will increase by approximately 2.89 kJ/kg [19]. It has been shown that long-term cold stress leads to an increase in feed intake, and more feed energy is used for thermogenesis to maintain the constancy of body temperature. However, digestive function is inhibited under cold stress, making the increased DMI unable to meet the increased heat production, leading to a decline in growth performance [3]. Kang et al. [12] reported that lower temperatures could lead to lower feed utilization and ADG in Korean cattle. Meanwhile, Zhang et al. [20] found that a cold environment induced a decrease in body weight as well as an increase in the expression of thermogenic genes in the liver of Mongolian sheep. From the data of the present study, it is likely that the increase in food intake in Simmental cattle during long-term cold stress is mainly used for resting thermogenesis to maintain body temperature, which causes the growth performance (body weight, ADG, feed utilization) to be inhibited.

Rectal temperature, respiratory rate, and pulse rate (heart rate) are important indicators for evaluating the internal environmental balance of animal organisms [7]. Lim et al. [21] observed a strong positive correlation between ambient THI values and rectal temperature (*R*^2^ = 0.901) and respiratory rate (*R*^2^ = 0.969). The rectal temperature (37.94 °C) and respiratory rate (20.53/min) of Sanhe cattle in winter are significantly lower than those in spring (38.41 °C; 27.07/min) [22]. Joy et al. [23] and Lees et al. [24] have conducted similar reports. However, the body’s heart rate is closely linked to metabolism, and increasing the heart rate can improve the body’s metabolic rate [3]. In this study, the heart rate of Simmental cattle in the W-CT group increased by 22.77 beats per minute compared to the A-ST group, and with the increase in the duration of cold stress, the heart rate also showed an increasing trend, which is similar to the research results of Li et al. [25]. In addition, cold stress also reduces the standing time and walking time of Simmental cattle, as standing increases the surface area of the body exposed to the wind and increases convective heat loss with the environment [4]. The above results indicate that under long-term cold stress, Simmental cattle can reduce heat dissipation by reducing respiratory frequency, standing time, and movement time and increase body heat production by increasing heart rate and DMI intake, thereby adapting the body to cold environments.

### 4.2. Changes in Rumen VFA and Apparent Digestibility of Nutrients

The pH value of rumen fluid is an important indicator reflecting the internal environment and fermentation degree of the rumen [26]. In this study, the pH value of ruminal fluid in the W-CT group increased, but there was no significant difference, consistent with the results of Kang et al. [12]. In addition, we observed a significant decrease in total VFA concentration under long-term cold stress; however, 75% of the ruminant’s energy requirement is derived from VFA [27]. The rumen flora is the main source of VFA and plays a major role in the degradation of feed in the rumen. The decrease in total VFA in the rumen under prolonged cold stress may result from a combination of changes in the rumen flora that decrease the amount of total VFA synthesized and the increase in VFA used to maintain body temperature. Meanwhile, some studies have shown that there is a weak negative correlation between the total VFA concentration and the pH value [28]. Therefore, in this study, the decrease in the total VFA concentration may be partially responsible for the slightly elevated pH value. As far as acetate and propionate are concerned, they mainly produce glucose through gluconeogenesis [29]. Kang et al. [12] and Guo et al. [30] found that acetate and propionate levels in the rumen decreased significantly when Korean cattle and sheep were subjected to long-term mild cold stress, and Guo et al. [30] also found that the abundance of *Prevotellaceac* in sheep rumen decreased. However, *Prevotellaceac* is usually a dominant bacterium in the rumen, whose main fermentation products are acetate and propionate [31,32]. Thus, the decrease in the concentration of acetate and propionate in the rumen of the W-CT group may be caused by the decrease in the abundance of *Prevotellaceac*. Propionate serves as a primary substrate for gluconeogenesis, and when ruminants are subjected to long-term intense cold stress, rumen microbes can absorb large amounts of propionate for gluconeogenesis [30], causing a significant increase in the acetate/propionate ratio.

Cold stress also enhances gastrointestinal contractions and peristalsis, feed exists within the gut for a shorter period of time, and microbes and digestive enzymes in the digestive tract do not sufficiently function such that feed is emptied without being thoroughly digested and absorbed, resulting in lower feed apparent digestibility [33]. Studies found that after cold stimulation, the ADG and the apparent digestibility of DM, CP, EE, Ca, and P in lambs significantly decreased [34,35]. In a low-temperature environment, the feed consumption and feed weight ratio of calves significantly increased, and the nutrient digestibility decreased [12,36]. This study found that long-term cold stress caused a decrease in the apparent digestibility of DM, CP, NDF, ADF, and energy in Simmental cattle, which was consistent with previous research results.

### 4.3. Changes in Biochemical Blood Indexes

Blood is the carrier for the transport of nutrients and metabolites within the body and is the pathway for the body to achieve humoral regulation. When the body is subjected to cold stress, the biochemical blood indexes (GLU, TG, hormones, etc.) can reflect the situation of the organic body’s energy metabolism. Coloma-Garcia et al. [37] found that blood GLU levels were increased in sheep under long-term cold stress. Liang et al. [38] suggested that the elevation of blood GLU content during cold stress could be used to enhance the body’s energy supply. Meanwhile, Hu et al. [9] also found that plasma propionate concentrations decreased significantly in both Holstein cows and Sanhe cattle when exposed to cold environments, suggesting that propionate may be a key source of the gluconeogenic process at the onset of cold stress. Furthermore, this study found that the plasma levels of CS, G-6-Pase, and PK in the W-CT group were significantly higher compared with those in the A-ST group. CS is the rate-limiting enzyme involved in the tricarboxylic acid cycle and regulates citrate levels [39]. G-6-Pase can release GLU into the blood through hydrolysis of glucose-6-phosphate in the liver and plays an important role in maintaining blood glucose homeostasis. Moreover, PK, as one of the main rate-limiting enzymes in the glycolysis process, can convert phosphoenolpyruvate and ADP into pyruvate and ATP [40]. These results suggest that when Simmental cattle are under long-term cold stress, the body’s glucose synthesis capacity and glycolytic activity are enhanced, which provides more energy to the body.

Changes in hormone levels are an important pathway for the organism to adapt to stress. The hormones involved in stress regulation include INS, GH, GC, thyroid hormones, etc. INS has the effect of enhancing anabolism and promoting energy deposition in the body [41]. Some studies have found that the content of insulin in the blood of cattle and sheep gradually decreases as the duration of cold stress increases [37,42]. Meanwhile, Sano et al. [43] also found that the concentration of glucagon (GCG) in the blood of sheep in cold environments (2 °C) was higher than that in the TNZ environment (20 °C). Although the levels of GCG were not examined in this study, due to the antagonism between GCG and INS, changes in GCG and INS levels are consistent with the fact that blood glucose concentrations increase. The primary role of GH is to promote organismal growth while also having anabolic effects. Bai’s [22] research shows that long-term cold stress can lead to obstacles in the synthesis and release of GH in dairy cows, affect the body’s anabolism, and inhibit the normal growth and development of dairy cows, which is consistent with the results of this study. GC is a stress hormone secreted by the adrenal cortex, which can promote liver gluconeogenesis and inhibit insulin-stimulated glucose uptake and utilization, as well as glycogen synthesis [44,45]. It has been reported that cold stress can activate the hypothalamus-pituitary-adrenal axis (HPA axis) in animals, promotes the release of GC, and cause an increase in blood glucose concentration [3]. Thyroid hormones mainly include T3 and T4, T4 is the main secretion of the thyroid gland, but it needs to be deiodinated to T3 to have biological activity. T3 can enhance tissue metabolism and increase ATP consumption in the body, thereby increasing body heat production [46]. Fukuharv et al. [47] reported that chronic cold stress increased blood levels of thyrotropin and thyroid hormones in rats. Meanwhile, Li et al. [48] found that the contents of T3 and T4 were significantly increased in Sanhe cattle under severe cold stress, which was consistent with the results of this study. The above results indicate that when Simmental cattle are subjected to long-term cold stress, the body can also promote the synthesis of GLU by regulating the contents of stress hormones, such as GC, T3, T4, INS, GCG, and GH, to meet the energy needs of the organism in cold environments. 

## 5. Conclusions

In summary, when Simmental cattle are under long-term cold stress, they can enhance the energy supply and heat production of the body by changing their physiological behavior, gastrointestinal function, and the content of enzymes and hormones to resist the challenges of cold environments, ultimately leading to retarded growth and development. Therefore, some measures are recommended to prevent wind exposure and keep warm in winter in order to reduce animal heat dissipation or improve nutrient digestibility and increase heat production by regulating rumen microbial composition, thereby reducing the impact of cold weather on the production performance of Simmental cattle.

## Figures and Tables

**Table 1:** TMR composition and nutrient composition (% of dry matter).

Ingredients	Content/%	Nutrient Composition	Content/%
Corn stalk	25.00	NEmf (MJ/kg)	6.89
Corn silage	25.00	Crude protein	12.80
Corn	25.00	Crude ash	8.64
Bran	8.00	Neutral detergent fiber	35.23
Soybean meal	7.00	Acid detergent fiber	19.95
Cottonseed meal	7.00	Acid detergent lignin	2.46
Urea	0.65	Hemicellulose	16.56
CaCO_3_	0.75	Cellulose	16.32
NaCl	0.50		
NaHCO_3_	0.35		
CaHPO_4_	0.25		
Premix ^1^	0.50		
Total	100.00		

^1^ The premix provided the following per kg of the diet: Fe 50 mg, Cu 10 mg, Mn 20 mg, Zn 30 mg, I 0.5 mg, Se 0.1 mg, VA 1500 IU, VD 550 IU, VE 10 IU.

**Table 2:** Effects of long-term cold stress on growth performance in Simmental bulls.

Item	A-ST	W-CT	*p*-Value
IBW, kg	348.47 ± 2.107	349.95 ± 2.187	0.630
FBW, kg	417.71 ± 3.934	392.85 ± 2.742	<0.001
ADG, kg/d	1.65 ± 0.068	1.02 ± 0.050	<0.001
DMI, kg/d	13.06 ± 0.399	14.40 ± 0.271	0.026
FE	8.18 ± 0.343	14.24 ± 0.490	<0.001

The results are presented as the mean ± SEM. Abbreviations: A-ST, autumn suitable temperature; W-CT, winter cold temperature; IBW, initial body weight; FBW, final body weight; ADG, average daily gain; DMI, dry matter intake; FE, feed efficiency = DMI/ADG.

**Table 3:** Effects of long-term cold stress on behavior in Simmental bulls.

Item	A-ST	W-CT	*p*-Value
Time, % of 24 h			
Lying, %	20.97 ± 0.357	26.18 ± 0.948	0.003
Lying rumination, %	18.47 ± 0.431	19.24 ± 0.404	0.139
Standing, %	23.68 ± 0.354	20.56 ± 0.731	0.005
Standing rumination, %	18.40 ± 0.792	15.83 ± 0.682	0.052
Feeding, %	12.01 ± 0.321	13.68 ± 0.281	0.021
Walking, %	5.28 ± 0.337	3.75 ± 0.202	0.005
Water drinking, %	1.18 ± 0.086	0.76 ± 0.070	0.006

The results are presented as the mean ± SEM. Abbreviations: A-ST, autumn suitable temperature; W-CT, winter cold temperature.

**Table 4:** Effects of long-term cold stress on rumen VFA in Simmental bulls.

Item	A-ST	W-CT	*p*-Value
pH	6.48 ± 0.028	6.56 ± 0.012	0.055
Acetate	60.77 ± 0.256	57.69 ± 0.291	<0.001
Propionate	16.70 ± 0.028	14.84 ± 0.111	<0.001
Butyrate	13.34 ± 0.091	13.13 ± 0.133	0.240
Total VFA	90.76 ± 0.291	85.66 ± 0.297	<0.001
Acetate:Propionate	3.64 ± 0.018	3.89 ± 0.031	<0.001

The results are presented as the mean ± SEM. Abbreviations: A-ST, autumn suitable temperature; W-CT, winter cold temperature.

**Table 5:** Effects of long-term cold stress on nutrients apparent digestibility in Simmental bulls.

Item	A-ST	W-CT	*p*-Value
DM	78.68 ± 0.363	76.57 ± 0.699	0.028
CP	71.33 ± 0.394	69.29 ± 0.481	0.011
NDF	57.54 ± 0.330	53.03 ± 0.316	<0.001
ADF	45.37 ± 0.487	41.16 ± 0.675	<0.001
Energy	71.25 ± 0.334	68.34 ± 0.427	<0.001

The results are presented as the mean ± SEM. Abbreviations: A-ST, autumn suitable temperature; W-CT, winter cold temperature; DM, dry matter; CP, crude protein; NDF, neutral detergent fiber; ADF, acid detergent fiber.

**Table 6:** Effects of long-term cold stress on plasma biochemical parameters in Simmental bulls.

Item	A-ST	W-CT	*p*-Value
GLU	4.51 ± 0.028	5.26 ± 0.022	<0.001
TG	0.29 ± 0.005	0.25 ± 0.008	0.004
NEFA	0.64 ± 0.005	0.62 ± 0.007	0.06
BHB	0.16 ± 0.003	0.13 ± 0.002	<0.001
Propionate	4.55 ± 0.042	3.89 ± 0.054	<0.001

The results are presented as the mean ± SEM. Abbreviations: A-ST, autumn suitable temperature; W-CT, winter cold temperature; GLU, glucose; TG, triglyceride; NEFA, non-esterified fatty acids; BHB, β-hydroxybutyrate.

**Table 7:** Effects of long-term cold stress on plasma stress hormones in Simmental bulls.

Item	A-ST	W-CT	*p*-Value
INS, ng/mL	1.20 ± 0.014	1.11 ± 0.014	<0.001
GH, ng/mL	23.03 ± 0.228	18.26 ± 0.130	<0.001
GC, ng/mL	0.19 ± 0.004	0.29 ± 0.004	<0.001
T3, ng/mL	4.36 ± 0.050	4.50 ± 0.040	0.025
T4, ng/mL	129.44 ± 0.502	141.60 ± 0.620	<0.001

The results are presented as the mean ± SEM. Abbreviations: A-ST, autumn suitable temperature; W-CT, winter cold temperature; INS, insulin; GH, growth hormone; GC, glucocorticoid; T3, triiodothyronine; T4, tetraiodothyronine.

## Data Availability

The data that support the findings of this study are available from the corresponding authors upon reasonable request.
